# A Test Case at Torquay

**Published:** 1914-02-14

**Authors:** 


### A Test Case at Torquay.

INFECTIOUS PATIENTS IN NURSING HOMES.

At Exeter on February 11, Judge Lush Wilson heard an action in which Miss Emmett, of the Pembroke Nursing Home, Clifton, claimed £48 damages from the Misses Camus and Hollis, of Kent House Nursing Home, Torquay, alleged to have been sustained by reason of the negligence of the defendants in permitting a nurse infected with measles to be removed from the Torquay Home to Pembroke Home, Clifton, without disclosing the fact that she had been in the house with infectious patients. Mr. Weathered appeared for the plaintiff and Mr. Hutchings for the defendants. After hearing the evidence, the Judge said that it was probably a dangerous thing to put infectious cases in a nursing home which might be crowded, but he knew no law against it. So far as he knew the law there was no legal liability in that case, he being unable to find that the defendants knew, or had reason to suspect, that the nurse was suffering from measles. Verdict for the defendants, with costs.

